# How professional logics shape AI implementation in mental healthcare: a qualitative study of an LLM-enhanced chatbot

**DOI:** 10.3389/fdgth.2026.1945281

**Published:** 2026-08-31

**Authors:** Silvia Rizzi, Stefano Fait, Mattia Franzin, Leonardo Sanna

**Affiliations:** 1 Digital Health Research, Centre for Digital Health & Wellbeing, FBK-Fondazione Bruno Kessler, Trento, Italy; 2 People Innovation for Research, FBK-Fondazione Bruno Kessler, Trento, Italy; 3 Digital Health Innovation Lab, Centre for Digital Health & Wellbeing, FBK-Fondazione Bruno Kessler, Trento, Italy; 4 Intelligent Digital Agents, Centre for Digital Health & Wellbeing, FBK-Fondazione Bruno Kessler, Trento, Italy

**Keywords:** digital mental health, general practice, healthcare professional logics, implementation science, large language models, LLM-enhanced chatbot, mental healthcare, primary care

## Abstract

**Introduction:**

Large language model (LLM)-enhanced chatbots are increasingly proposed as scalable tools for mental health support. However, their successful integration into healthcare depends not only on technical performance, but also on clinicians' acceptance of these technologies and their perceptions of the value these tools can bring to clinical practice. This study examined how psychotherapists and general practitioners (GPs) perceived the potential of the implementation of an LLM-enhanced psychoeducational chatbot in routine mental healthcare.

**Methods:**

We conducted a qualitative, practice-informed study using four profession-specific focus groups with 48 clinicians in Italy, including 40 psychotherapists and 8 GPs. Participants directly interacted with a digital prototype based on the World Health Organization's Self-Help Plus (SH+) intervention, in which structured psychoeducational content was combined with constrained LLM-based conversational support. Data included participants' written reflections, structured group outputs, and researchers' field notes. Materials were analyzed using inductive thematic analysis.

**Results:**

Across both professional groups, participants evaluated the chatbot primarily in relation to its place within care pathways, its appropriateness for different users, the level of professional oversight required, and the risks associated with its use. Two distinct professional logics emerged. Psychotherapists framed the chatbot as a clinician-guided adjunct to psychotherapy, valuing it for therapeutic continuity while emphasizing the need for contextualization and supervision. GPs framed it as a low-threshold preventive and signposting resource, valuing accessibility and feasibility within primary care while emphasizing clear eligibility criteria and referral pathways. Although both groups identified similar safety concerns, they proposed different mitigation strategies, leading to distinct implementation models.

**Conclusion:**

The findings suggest that the implementation of LLM-enhanced mental health chatbots is shaped by profession-specific logics rather than by a single, uniform model of adoption. Successful integration therefore requires context-sensitive governance and implementation strategies tailored to different care settings, professional responsibilities, and risk management assumptions.

## 1 Introduction

Large language models (LLMs) are redefining the capabilities of digital health technologies. Recent reviews suggest that LLM-based applications are rapidly expanding across mental health support, assessment, psychoeducation, and conversational care contexts (1, 2). By enabling natural, context-sensitive, and adaptive interactions, they have substantially expanded the potential of conversational agents to support health education, self-management, patient engagement, and mental healthcare. In mental health, LLM-enhanced chatbots have emerged as promising tools to address persistent gaps in access to psychological support, offering scalable interventions that may complement existing services and extend care beyond traditional clinical settings. Recent reviews and meta-analytic work suggest growing evidence for the relevance of chatbot-based and generative AI interventions in mental health, while also highlighting substantial heterogeneity in design, target populations, and evidence quality (3–6). Recent advances in generative AI have further strengthened this promise by enabling conversational interactions often perceived as more empathic, coherent, and responsive, while at the same time raising questions about the appropriate clinical boundaries of such systems (7–10).

Yet, as AI technologies mature, the principal challenge confronting digital mental health is changing. The field has invested considerable effort in demonstrating that conversational agents can be usable, acceptable, and clinically beneficial under appropriate conditions. Increasingly, however, the limiting factor is not technological capability but the successful implementation and routine use of these systems in real-world healthcare settings. This shift reflects a broader transition in digital mental health research from demonstrating efficacy or acceptability toward understanding service integration, organizational fit, and sustainable models of care (11). Whether AI ultimately improves healthcare depends less on what these systems are technically capable of doing and more on whether they can be meaningfully incorporated into routine clinical practice. Successful implementation requires alignment with organizational structures, professional responsibilities, governance mechanisms, clinical workflows, and models of care. Consequently, the future impact of LLM-enabled interventions is likely to be determined as much by healthcare organizations and professional practice as by further advances in artificial intelligence itself.

The successful implementation of AI in healthcare depends on aligning AI systems with the human processes through which clinical work is organized, responsibility is allocated, and care is delivered. Clinical decisions are shaped by multiple sources of knowledge and authority. Increasingly, these may include algorithmic outputs alongside professional judgment, organizational routines, established care pathways, and systems of accountability.

This perspective is consistent with implementation science, which conceptualizes technology adoption as a sociotechnical process rather than a purely technical one. Frameworks such as the Non-adoption, Abandonment, Scale-up, Spread and Sustainability (NASSS) framework emphasize that successful implementation emerges from interactions among technological characteristics, organizational contexts, professional work, and wider health system dynamics (12). Technologies are therefore not simply introduced into healthcare settings; they are actively interpreted, negotiated, and reshaped through everyday clinical practice. Their function is not fixed by design but co-constructed through the routines, responsibilities, and institutional arrangements that govern healthcare delivery (13).

From this perspective, implementation is not simply about deploying a mature, technically functioning technology into healthcare. Rather, it is the process through which healthcare professionals collectively define what the technology is for, who should use it, under which conditions, and with what forms of responsibility and oversight. AI systems therefore become part of routine clinical practice only after healthcare professionals assign them a socially and professionally legitimate role within existing models of care.

The role of healthcare professionals is particularly critical in this process. Sociological theories of the professions argue that professional groups do not merely perform different tasks; they operate according to distinct professional logics that define legitimate knowledge, appropriate clinical action, responsibility, and acceptable standards of care. From this perspective, introducing AI is not simply a matter of deploying a new technological tool; it requires aligning the technology with existing clinical roles, responsibilities, and models of care. The same AI system may therefore acquire fundamentally different roles depending on how different professional communities interpret its place within patient care. This is consistent with recent healthcare research showing that practitioners navigate technologies and organizational change through competing or coexisting institutional and professional logics (14, 15).

Mental healthcare represents an especially informative context in which to examine these dynamics. Unlike many other healthcare domains, mental health care is deeply relational, relies on contextual interpretation and longitudinal engagement, and requires continuous management of uncertainty, vulnerability, and risk. Consequently, integrating conversational AI into mental health services raises questions that extend well beyond usability or technical performance. Mental healthcare is a particularly revealing context for AI implementation because support is relational, context-sensitive, and risk-laden, making appropriateness, escalation, and accountability central design and implementation concerns (10, 16, 17). Decisions regarding whether an LLM-enhanced chatbot should function as an autonomous self-help resource, a clinician-supervised intervention, or a gateway to specialist care are therefore not merely design choices; they reflect fundamentally different conceptions of appropriate care.

Despite the rapid growth of digital mental health research, existing reviews suggest that the literature remains heavily oriented toward intervention design, performance, user outcomes, and acceptability, with far less attention to how clinicians and different professional groups interpret implementation in routine care (1, 6). Therefore, the perspectives of healthcare professionals responsible for adopting and integrating these technologies into routine practice remain underexplored. Beyond this general gap, even fewer studies have examined whether different professional groups construct different clinical meanings around the same AI technology. This omission is significant because implementation strategies frequently assume a relatively homogeneous healthcare workforce, overlooking the possibility that distinct professions may evaluate AI through different clinical priorities, organizational constraints, and professional responsibilities.

We address this gap by conceptualizing AI implementation as a process of professional sensemaking. Rather than asking whether healthcare professionals accept conversational AI, we investigate how they perceive its clinical role, value, and appropriate integration within mental healthcare. Specifically, we explore how psychotherapists and general practitioners perceive the potential integration and clinical role of an LLM-enhanced psychoeducational chatbot based on the World Health Organization's Self-Help Plus (SH+) intervention in mental healthcare (18). Through profession-specific qualitative focus groups following direct interaction with the system, we examine how clinicians position the chatbot within existing care pathways, negotiate professional responsibilities, identify implementation risks, and articulate the organizational conditions required for its sustainable integration.

Our findings reveal that the same technological artefact gives rise to two coherent but distinct professional logics. Psychotherapists predominantly conceptualize the chatbot as a clinician-guided extension of psychotherapy, emphasizing therapeutic continuity and clinical oversight. In contrast, general practitioners primarily frame it as a low-threshold preventive and signposting resource that facilitates early access to mental health support within primary care. These contrasting interpretations generate different expectations regarding governance, accountability, workflow integration, and risk management, suggesting that implementation cannot be understood as a universal process independent of professional context.

This study contributes to the digital health literature in three ways. First, it shifts attention from technology acceptance toward professional sensemaking as a key mechanism shaping AI implementation. Second, it demonstrates that differences between healthcare professions should be understood not merely as barriers to adoption but as distinct professional logics that generate alternative implementation models. Third, it argues that the successful integration of LLM-enhanced mental health technologies depends on designing implementation strategies that align with the organizational realities and professional jurisdictions of different care settings. More broadly, the study suggests that the future of AI in healthcare will depend not only on increasingly capable algorithms but also on understanding how healthcare professionals collectively define the legitimate place of AI within clinical practice. Accordingly, this study examines how psychotherapists and general practitioners define the legitimate role of an LLM-enhanced psychoeducational chatbot in mental healthcare, and how these profession-specific interpretations shape perceived risks, governance expectations, and implementation requirements.

## 2 Methods

We conducted a qualitative, practice-informed study using profession-specific focus groups grounded in direct interaction with a digital SH + prototype. The study aimed to explore how psychotherapists and general practitioners (GPs) understood the potential role, limits, and implementation requirements of an LLM-enhanced mental health chatbot within their respective care settings.

This design aligns with emerging evidence from clinical and real-world evaluations suggesting that chatbot-based mental health interventions can support user engagement and acceptability over time, including in randomized controlled studies (3, 4, 19). It is also consistent with empirical findings indicating that LLM-generated responses may be perceived as more empathic or comparable in empathy to human-authored responses in controlled settings (7, 8). At the same time, the literature highlights important safety considerations, methodological limitations, and ongoing challenges in aligning evaluation metrics with clinically meaningful and user-relevant outcomes in conversational agents for mental health (20).

### 2.1 Study design and theoretical framework

A qualitative, practice-informed design was selected because the study aimed to investigate how clinicians interpreted the potential place of an LLM-enhanced chatbot within existing care pathways, professional responsibilities, and organizational routines, rather than to measure technology acceptance quantitatively.

The study was informed by implementation science and sociotechnical perspectives on healthcare innovation, particularly the Non-adoption, Abandonment, Scale-up, Spread, and Sustainability (NASSS) framework (12). The analytical orientation combined inductive thematic coding of clinicians' immediate reactions with an abductive synthesis to interpret how empirical findings mapped onto broader sociotechnical and professional dimensions. Reporting was guided by the Consolidated Criteria for Reporting Qualitative Research (COREQ), and the completed checklist is provided as Supplementary Material S1. To further enhance analytic transparency, Supplementary Table S1 maps the final themes to the three data sources used in the analysis.

### 2.2 Participants and recruitment

Participants were recruited through convenience sampling from two professional groups involved in mental health support and referral within the local healthcare system of the Autonomous Province of Trento, Italy (Table 1). The first group comprised licensed psychotherapists (*n* = 40) affiliated with the local health authority (Azienda Sanitaria Universitaria Integrata del Trentino, ASUIT) who chose to participate in a 3 h workshop entitled *Digital therapies and AI to support the work of psychologists: guided experimentation and clinical reflection*, organized by Fondazione Bruno Kessler. In the Italian context, the professional title psychotherapist refers to a practitioner who has completed a degree in psychology or in medicine and surgery followed by recognized specialist training in psychotherapy of at least four years; psychotherapists without a medical degree are not authorized to provide purely medical interventions. Because the present study did not systematically collect participants' original degree background, we cannot distinguish within the sample between psychotherapists with psychology vs. medical training. These clinicians operated across public mental health outpatient clinics, community centers, and specialized child/adolescent neuropsychiatry services. To explore nuances across practice areas, the 40 psychotherapists were organized into three parallel, profession-specific focus groups: one working predominantly with children and adolescents (*n* = 13), one working with adult populations (*n* = 14), and one mixed clinical practice group (*n* = 13).

The second group comprised general practitioners (GPs, *n* = 8) practicing within the National Health Service in the primary care district of Tione (Trentino, Italy), who participated in a 2 h dedicated focus group session. These clinicians operated as first-contact, panel-based primary care physicians managing acute and chronic distress and coordinating patient referrals.

**Table 1:** Characteristics of the participating clinicians and composition of the profession-specific focus groups.

Professional group	N	N. of FG	Group composition	Recruitment context	Role in care
Licensed psychotherapists (Italian professional qualification)	40	3	1 group working with children and adolescents, 1 with adults, and 1 mixed group	Licensed psychotherapists affiliated with the local health authority (ASUIT)	Professionals operating within relational, longitudinal, and clinically intensive models of psychological care
General practitioners (GPs)	8	1	1 profession-specific GPs FG	GPs currently involved in patient care in the area of Tione, Italy	Primary care physicians operating within a high-volume, panel-based population model
Total	48	4	Profession-specific groups only	Convenience sampling	Clinicians with direct experience in mental health support and referral processes

Demographic and additional professional characteristics were not systematically collected for the present study and are therefore not reported. In Italy, the title psychotherapist indicates a professional who has completed a degree in psychology or medicine and surgery followed by recognized specialist psychotherapy training. The present study did not systematically collect participants’ original degree background and therefore does not distinguish within the sample between psychotherapists with psychology vs. medical training.

While participants regularly utilized routine electronic medical records in clinical practice, none reported prior structured clinical experience with LLM-enhanced or generative AI tools in therapeutic workflows. Individual demographic metrics (such as exact age and years in practice) were not systematically recorded to preserve anonymity in an institutional workshop setting; however, participants represented a broad cross-section of early-, mid-, and senior-career professionals.

The two professional groups represented distinct positions within mental healthcare pathways. In the study context, psychotherapists were more closely associated with relational, longitudinal, and clinically intensive models of psychological care, whereas GPs operated within a primary care setting characterized by first-contact access, broad patient panels, and the need to make decisions under conditions of limited time and high service demand. The study was not designed as a numerically symmetrical comparison between professions, but to explore how these different professional contexts shaped interpretations of the same technological artifact.

### 2.3 The technological artefact

The discussions were anchored in direct interaction with a digital prototype based on the World Health Organization's Self-Help Plus (SH+) intervention. The prototype represented a digital adaptation of SH+, a transdiagnostic, low-intensity psychological intervention developed for scalable delivery across different cultural and healthcare settings.

The system was designed as a hybrid conversational agent intended to balance user engagement with clinical safety. The psychoeducational and therapeutic content remained fully structured and protocol-driven in order to preserve intervention fidelity and prevent the system from autonomously modifying clinical content. LLM-based functionality was integrated only to support relational and conversational features, such as dialogue flow, perceived naturalness, and empathic tone. These LLM features were explicitly constrained and bounded by technical guardrails so that the system could not provide autonomous diagnostic or therapeutic advice.

During the sessions, participants were asked to test the introductory module of the intervention (Session 0: Welcome and Onboarding) and the first intervention module (Session 1: Grounding) using their own smartphones.

### 2.4 Data collection procedure

Data collection took place through profession-specific focus groups embedded within a structured workshop format. Focus group discussions were deliberately not audio- or video-recorded to ensure a low-stakes, non-evaluative environment that encouraged candid, uninhibited reflection on institutional liabilities, professional boundaries, and clinical uncertainties, as well as to adhere to institutional workshop agreements. To ensure robust data triangulation without verbatim audio recording, data were systematically captured through three complementary sources:

1. Individual Written Reflection Sheets (*n* = 48): immediately following a 20 min hands-on testing phase where each clinician interacted with the SH+ prototype on their smartphone, participants completed an individual sheet documenting immediate observations on usability, perceived conversational empathy, tone boundaries, and points requiring clinical escalation.
2. Structured Small-Group Worksheets (*n* = 11): participants then convened in small groups of 3 to 5 within their professional cohort (yielding 9 worksheets from psychotherapists and 2 from GPs). Each group collaboratively completed a structured worksheet addressing target clinical populations, exclusion criteria, optimal placement within care pathways, clinician oversight requirements, and identified safety risks with corresponding mitigation strategies.
3. Structured Researcher Field Notes: a designated researcher (LS) took comprehensive, real-time observational field notes throughout the small-group discussions and plenary debriefings (generating 14 pages of single-spaced notes). Notes were structured using a standardized observational guide focusing on points of consensus, intra-group debates, and organizational requirements.

Each session concluded with a facilitated plenary discussion (led by SR, a clinical psychologist and researcher). This plenary debriefing served as an immediate communicative validation (member-checking) phase, where synthesized impressions were reflected back to the participants for real-time clarification, refinement, and endorsement.

This design was intended to elicit not only immediate reactions to the tool, but also participants' views on clinical appropriateness, implementation risks, professional responsibility, and organizational requirements for safe use.

Verbatim excerpts are identified by professional group and source type (e.g., PT-WR = psychotherapist written reflection; PT-GW = psychotherapist group worksheet; GP-WR = GP written reflection; GP-GW = GP group worksheet).

### 2.5 Ethics

According to Italian Law No. 3/2018, formal ethical approval was not required for this study. All participants were informed about the purpose of the study, the voluntary nature of participation, and the handling of anonymized data. Informed consent was obtained from all participants prior to participation.

### 2.6 Data analysis

The dataset was analyzed using inductive thematic analysis. Analysis focused on how participants interpreted the perceived role and function of the chatbot, its appropriate users and boundaries of use, the role of the clinician, the main implementation risks, and the organizational conditions required for safe and legitimate integration into care pathways.

The analysis proceeded in five distinct stages:

1. Familiarization: all written reflections (*n* = 48), structured group worksheets (*n* = 11), and field notes were transcribed into digital format and read repeatedly by SR, LS, and SF to achieve comprehensive dataset immersion.
2. Initial Open Coding: Two researchers (SR, LS) independently performed open qualitative coding across the textual corpus, assigning descriptive codes to discrete meaning units related to clinical utility, perceived risk, workflow fit, and governance. This initial inductive phase generated a pool of 54 primary codes.
3. Code Clustering and Refinement: The primary codes were compared, merged, and organized into 16 s-order subthemes using collaborative digital spreadsheets and concept mapping tools. Coding discrepancies between SR and LS were examined in reflexive meetings with a third researcher (SF) and resolved through team consensus.
4. Thematic Conceptualization: The refined subthemes were then abductively mapped onto the theoretical dimensions of professional logics and sociotechnical implementation. Themes were analyzed both within each professional group (to establish internal coherence) and across groups (to identify contrasting implementation models).
5. Information Power and Review: Given the exploratory nature of the study, formal *post-hoc* data saturation was not claimed. Instead, sampling adequacy was evaluated. The study's narrow aim, dense participant expertise, theoretically grounded inquiry, and rich interactive prototype data provided strong information power to delineate distinct professional logics.

Consistent with qualitative methodology, the findings are intended to offer context-sensitive, transferable insights into professional sensemaking rather than statistically generalizable population traits.

### 2.7 Researcher reflexivity

The research team brought together perspectives from psychology, digital health, and AI development. SR, who facilitated the sessions, is a psychologist and researcher, while the wider team included researchers working on digital health innovation and intelligent digital agents. This interdisciplinary positioning supported informed facilitation of the prototype sessions and interpretation of implementation issues, but it may also have shaped the aspects of the technology that were foregrounded during data collection and analysis. To reduce this risk, interpretations were discussed across team members with different disciplinary perspectives, and particular attention was paid to concerns, restrictions, and less favorable views expressed in the materials rather than only to implementation opportunities.

## 3 Results

The analysis drew on participants' written reflections during prototype testing, structured small-group outputs, and researchers' field notes from the facilitated discussions. Across these materials, participants did not primarily evaluate the SH+ digital prototype as a neutral technological artefact or in terms of technical performance alone. Rather, they assessed it in relation to its place within existing care pathways, its appropriateness for different users, the level of professional oversight required, and the risks associated with its use in practice.

Within this shared evaluative frame, two distinct professional logics emerged. Psychotherapists predominantly positioned the chatbot as a clinician-guided adjunct to psychotherapy, whereas general practitioners (GPs) more often framed it as a low-threshold preventive and signposting resource for use within primary care. These profession-specific interpretations were associated with different expectations regarding patient selection, governance, workflow integration, and risk management.

The presence of the main themes across the three data sources is summarized in Supplementary Table S1.

### 3.1 Shared evaluative criteria for acceptable use

Across both professional groups, participants assessed the prototype through a common set of practical and clinical criteria. In their written reflections and group discussions, they paid particular attention to the clarity of the instructions, the usability of the proposed exercises, the relevance of the content to everyday life, and the extent to which the conversational support appeared coherent, bounded, and helpful. For example, one psychotherapist wrote that “The tool could be useful, but only if its scope is very clearly defined and users understand what it is for and what it is not for” (PT-WR). Similarly, a GP worksheet emphasized that “the main issue is not whether the chatbot works in general, but where exactly it fits in the care pathway” (GP-WR). These dimensions were not treated as merely technical features, but as conditions influencing whether the intervention could be regarded as legitimate and acceptable within routine care.

A recurrent concern across materials was the appropriateness of use. Participants in both groups questioned for whom the tool might be suitable, where its limits should be drawn, and at what point self-help support would become insufficient. Acceptability was therefore linked less to general enthusiasm for digital innovation than to judgments about fit with existing professional responsibilities, thresholds of clinical need, and the practical conditions under which the chatbot might be used safely.

### 3.2 Psychotherapists: a clinician-guided adjunct model

Psychotherapists predominantly understood the chatbot as potentially useful when embedded within an ongoing therapeutic process. One participant described it as “something that supports the work between sessions, but not as something that replaces the therapeutic process” (PT-GW), while another noted that “its role makes sense only if it is embedded in an ongoing therapeutic relationship” (PT-WR). In this framing, the tool was valued for its capacity to reinforce content already introduced in therapy, support reflection between sessions, encourage practice, and contribute to continuity between clinical encounters. Its usefulness was thus tied to its role as an adjunct to psychotherapy rather than as a stand-alone intervention.

At the same time, psychotherapists repeatedly highlighted the importance of clinical contextualization. Across written comments and field notes, they questioned whether the chatbot could respond appropriately to emotionally complex, ambiguous, or clinically sensitive situations without access to the broader therapeutic formulation. What mattered was not only the quality of the chatbot's responses, but whether these remained within the boundaries of supportive psychoeducation and did not drift into individualized clinical guidance.

Minor operational nuances emerged across the psychotherapist subgroups. While adult-focused therapists emphasized the chatbot's capacity to reinforce cognitive-behavioral homework and therapeutic continuity between weekly sessions, practitioners working with children and adolescents placed greater emphasis on developmental readability, the risk of unmonitored digital reliance, and the indispensable need to involve parents and caregivers in the onboarding and oversight process.

Within this professional logic, the chatbot was considered acceptable primarily when its function remained explicitly bounded and professionally supervised. Psychotherapists therefore tended to envisage implementation models in which the tool would be introduced by the clinician, linked to therapeutic goals, and used under conditions of ongoing interpretive oversight.

### 3.3 GPs: a low-threshold preventive and signposting model

GPs, by contrast, more often framed the chatbot as an accessible first-line support resource for patients experiencing distress that did not necessarily require specialist psychotherapy. As one participant wrote, “This could be useful as a first step for people who are distressed but not yet in specialist care” (GP-WR), while a group worksheet emphasized that “its value is in offering early support and helping identify when more structured care is needed” (GP-GW).

In their reflections, the tool was positioned less as part of a therapeutic relationship and more as a practical option for early support, orientation, and structured self-help. Its value lay in offering something immediate, concrete, and scalable within the constraints of primary care.

This framing emphasized low-threshold access and the possibility of recommending the tool to patients who might benefit from psychoeducational support, activation, or guided self-management, particularly in contexts where specialist services were not immediately available. Rather than focusing on detailed therapeutic integration, GPs were more likely to evaluate the chatbot in terms of feasibility, accessibility, and potential usefulness within a broader stepped-care or referral pathway.

At the same time, this relative openness was accompanied by an emphasis on clear boundaries of use. Safety was commonly framed in terms of selecting appropriate users, avoiding use in more severe or complex situations, and ensuring that escalation or referral options were available when needed. In this perspective, the chatbot was located at the margins of treatment rather than within the core of an ongoing clinical relationship.

### 3.4 Shared concerns about risk, divergent mitigation strategies

Concerns about risk were visible in both groups. One psychotherapist warned that “there is a risk that people may rely on it too much and delay seeking professional help when they actually need it” (PT-WR), whereas GP participants emphasized the importance of excluding “signs of crisis, severe symptoms, or situations that require immediate clinical assessment” (GP-W).

Although both groups identified safety and risk as central issues, they proposed different ways of addressing them. Across the materials, participants raised concerns about ambiguous responses, excessive apparent certainty, and the possibility that the conversational component might be used in situations requiring more direct or individualized clinical assessment. These concerns were especially salient in relation to the LLM-based support, which participants expected to remain helpful and motivating without exceeding its intended function. Psychotherapists more often emphasized supervision and contextual interpretation, for example noting that “the safest way to use it would be within a therapeutic pathway where someone can monitor how the patient is engaging with it” (PT-GW). Their proposed safeguards centered on tighter integration with psychotherapy, clearer clinician oversight, and more explicit limits on autonomous use. GPs, by contrast, more often approached safety through boundary-setting and triage, emphasizing the importance of eligibility criteria, scope restriction, and clearly defined pathways for escalation or referral, with one worksheet stating that “the key issue is having clear eligibility criteria and a clear next step if the situation goes beyond what the tool can handle” (GP-GW).

### 3.5 From prototype evaluation to implementation requirements

Participants' evaluations extended beyond immediate impressions of the tool and frequently developed into broader reflections on implementation. Across the structured group outputs and plenary discussions, the acceptability of the chatbot was linked to organizational requirements such as clarity of professional responsibility, procedures for identifying inappropriate use or deterioration, and the availability of defined pathways for escalation or referral. The central question was not simply whether the tool worked, but under which organizational conditions its use could be considered legitimate and safe.

These requirements were themselves shaped by the two professional logics. For psychotherapists, implementation implied stronger integration with therapeutic workflows and clearer interpretive accountability, as one participant put it, “someone has to remain clearly responsible for when the chatbot is introduced, how it is used, and when it is no longer appropriate” (PT-GW). For GPs, implementation implied feasibility within routine primary care, ease of recommendation, and clearly bounded use within a wider service network: “there must be a clear step-up procedure for cases that exceed the intended level of support” (GP-GW), indicating that implementation was understood in organizational as well as technical terms. In both cases, participants framed implementation as a sociotechnical issue requiring alignment between the function of the tool and the care setting in which it would be deployed.

### 3.6 Two professional logics, two implementation models

Taken together, the findings suggest that the same technological artifact gave rise to two distinct implementation models (Table 2). Psychotherapists framed the chatbot as a tightly bounded adjunct to psychotherapy, whose legitimacy depended on therapeutic integration, clinical supervision, and continuity of care. GPs, in contrast, framed it as a preventive and signposting resource, suitable for low-intensity support and early orientation outside specialist treatment settings.

**Table 2:** Contrasting professional logics shaping the implementation of an LLM-enhanced chatbot in mental healthcare.

Theme	Psychotherapists	GPs	Implication
Primary lens of evaluation	Evaluated the chatbot in relation to psychotherapy processes, continuity of care, and clinical meaning-making	Evaluated the chatbot in relation to accessibility, early support, and feasibility within routine primary care	The tool is interpreted through setting-specific models of care rather than as a neutral technology
Perceived role of the chatbot	Clinician-guided adjunct to psychotherapy	Low-threshold preventive and signposting resource	The same tool is assigned different clinical roles across professions
Expected clinical function	Reinforce therapeutic content, support reflection between sessions, extend work beyond encounters	Provide first-line support, structured self-help, orientation, and possible bridge to further care	Deployment models should reflect different intended functions rather than assume uniform use
View of acceptable use	Acceptable when bounded, supervised, and linked to therapeutic goals	Acceptable when targeted to appropriate users and embedded in clear step-up/referral pathways	Acceptability depends on contextual fit with professional responsibilities
Main concern	Loss of contextual interpretation in complex or emotionally sensitive situations	Inappropriate use with patients requiring more intensive or specialized care	Risk is defined differently according to care setting and professional responsibility
Approach to safety	Safety through clinical containment, therapeutic oversight, and restricted autonomy	Safety through boundary-setting, eligibility criteria, triage, and escalation pathways	Safety is not only a technical property but an organizational achievement
Role of the clinician	Active interpretive supervisor who introduces, frames, and monitors use	Gatekeeper who recommends, filters, and redirects patients when needed	Governance models should specify different forms of clinician involvement
Preferred implementation setting	Within or alongside ongoing psychotherapy	Within primary care as part of low-intensity support or referral infrastructure	One-size-fits-all implementation is unlikely to be effective
Governance needs	Clear supervision, therapeutic integration, and accountability for use within treatment	Clear scope of use, criteria for appropriateness, and referral/escalation mechanisms	Governance should be tailored to professional workflow and organizational context
Overall implementation model	Tightly bounded adjunct model	Preventive and signposting model	Implementation strategies should be profession-sensitive and setting-specific

These differences were not simply variations in personal preference. Rather, they reflected deeper distinctions in how each group understood appropriate care, professional responsibility, and the role of digital tools in mental health support. The findings therefore indicate that the successful integration of LLM-enhanced mental health chatbots is unlikely to follow a single standardized pathway. Instead, implementation strategies need to be tailored to the professional logic, workflow, and risk management assumptions of different care settings.

Overall, participants' responses suggest that the key issue was not whether AI-supported mental health tools are acceptable in principle, but how their role is defined in relation to existing models of care, professional accountability, and service organization.

## 4 Discussion

This study explored how psychotherapists and general practitioners (GPs) conceptualized the role and implementation of an LLM-enhanced psychoeducational chatbot in mental healthcare after direct interaction with a digital SH+ prototype. Rather than revealing simple differences in attitudes toward AI, the findings suggest that the same technological artifact was interpreted through distinct professional logics that assigned different clinical purposes, responsibilities, and implementation requirements to the tool. In this sense, the study indicates that AI implementation in mental healthcare is not only a matter of technical capability or adoption readiness, but also a process of professional sensemaking. This interpretation is consistent with broader work on sociotechnical implementation and human-AI collaboration in healthcare, which shows that the clinical value of AI depends on task division, oversight, workflow integration, and institutional role definition (21–23).

This finding contributes to current research on conversational AI in mental health, which has largely focused on usability, engagement, effectiveness, and ethical considerations. These lines of work are essential, but they often imply that once a chatbot demonstrates sufficient technical and clinical promise, implementation becomes primarily a matter of scaling or adoption. Our findings suggest a different interpretation. Participants did not primarily assess the chatbot as an isolated technological product. Instead, they evaluated it in relation to care pathways, thresholds of clinical need, professional accountability, and the organizational conditions under which it could be used safely and legitimately. The key question for participants was therefore not simply whether the chatbot worked, but what kind of clinical role it should occupy within the healthcare system.

Our findings suggest that an important challenge for AI implementation and adoption in healthcare is not only improving system performance, but also understanding how AI systems can be incorporated into everyday clinical practice. Healthcare professionals did not evaluate the chatbot solely in terms of its technical capabilities; rather, they considered how it could be positioned within existing care pathways, professional responsibilities, organizational routines, and models of care. Questions regarding who should use the technology, under which conditions, and who should retain responsibility for decisions were central to participants' reflections. Therefore, implementation should be understood not only as a process of technological deployment, but also as a process through which new tools become integrated into professional practices and organizational contexts.

This perspective aligns with implementation science, which conceptualizes the introduction of health technologies as a sociotechnical process shaped by interactions among technical systems, organizational contexts, professional work, and service structures. From this perspective, implementation does not consist in inserting a stable technology into a neutral clinical environment. Rather, technologies acquire meaning through the ways in which they are interpreted, bounded, and incorporated into existing models of care. Our findings suggest that this process is strongly mediated by professional logics. The chatbot did not have a single self-evident function; instead, its potential role was actively reconstructed in relation to the distinct assumptions, responsibilities, and workflows of different professional groups.

The contrast between psychotherapists and GPs is especially informative in this regard. Psychotherapists predominantly interpreted the chatbot through a logic of therapeutic continuity. This interpretation is compatible with blended or hybrid models of mental health care, in which digital tools are not positioned as stand-alone substitutes but as structured extensions of clinician-led treatment (11, 24). Within this framing, the tool was considered potentially useful insofar as it could reinforce content already introduced in therapy, support reflection between sessions, and extend therapeutic work beyond face-to-face encounters. However, this potential value remained conditional on clinical contextualization and professional oversight. Psychotherapists were concerned that emotionally complex or ambiguous situations could not be adequately managed without access to the broader therapeutic formulation. As a result, they tended to view the chatbot as acceptable only when functioning as a tightly bounded adjunct to psychotherapy rather than as a stand-alone intervention.

GPs, by contrast, more often interpreted the same tool through a logic of low-threshold access and population-oriented primary care. This framing is consistent with stepped-care and primary-care-oriented approaches, in which low-intensity digital resources may serve as early support, self-management aids, or bridges to more intensive care when needed (25–27). Within this framing, the chatbot was seen less as an extension of an ongoing therapeutic relationship and more as a practical resource for early support, structured self-help, and signposting within routine primary care. Its perceived value lay in accessibility, immediacy, and the possibility of offering some form of support in contexts where specialist psychological care might not be readily available. This did not mean that GPs were unconcerned about risk. Rather, their approach to safety was shaped by the practical realities of primary care and by the need to define eligibility, scope, and escalation routes clearly.

Importantly, these profession-specific framings generated different implementation models. For psychotherapists, implementation implied integration into therapeutic workflows, clinician introduction of the tool, ongoing interpretive oversight, and clear limits on autonomous use. For GPs, implementation implied feasibility within routine primary care, ease of recommendation, low-threshold accessibility, and clearly defined referral or escalation pathways. The same technological artifact therefore gave rise to different organizational requirements depending on the professional context in which it was being situated. This suggests that implementation strategies for LLM-enhanced mental health tools are unlikely to be effective if designed as one-size-fits-all deployment models. More broadly, recent digital mental health scholarship suggests that implementation success depends on matching intervention intensity, delivery model, and clinician involvement to the care setting and target population (11, 24, 28).

A further important finding concerns the meaning of safety in participants' accounts. In our data, safety concerns did not remain at the level of abstract discussion about AI risk. Instead, they were articulated through practical questions about who should use the chatbot, under what conditions, how emotionally sensitive or clinically complex situations should be recognized, and what forms of supervision or escalation should be available. Psychotherapists tended to translate these concerns into a need for tighter clinical containment and interpretive oversight, whereas GPs more often emphasized eligibility criteria, scope restriction, and referral pathways. In this sense, safety emerged not simply as a technical property of the system, but as something participants understood in relation to professional responsibility and service organization. This supports emerging arguments that safe AI in healthcare cannot be secured by model performance alone, but also requires governance frameworks that specify oversight, escalation, responsibility allocation, and context-appropriate use (29–31).

This finding has direct implications for governance. In the present study, governance was not primarily discussed in terms of abstract regulation, but in terms of practical implementation arrangements: who introduces the tool, who remains accountable for its use, when autonomous use becomes inappropriate, and how step-up procedures should be activated. Our findings therefore suggest that governance for LLM-enhanced mental health tools must be grounded in empirically identifiable care pathways and professional roles, rather than assumed to follow from model-level safeguards alone (21, 23). However, participants' responses suggest that governance must also address implementation-level questions, including responsibility allocation, patient selection, scope of use, escalation procedures, and the integration of AI tools within existing care pathways. In practice, this means that trustworthy AI in mental healthcare depends not only on how systems are designed, but also on how their role is institutionally defined and operationalized.

The findings also speak to how professional heterogeneity is understood in digital health implementation. Differences between professions are often treated as barriers to adoption or as resistance to innovation that must be overcome through training or change management. Our findings suggest a more constructive interpretation. The contrasting views expressed by psychotherapists and GPs can be understood as valuable forms of implementation knowledge because they reveal the different conditions under which the same technology may become clinically legitimate (14, 23). Rather than aiming to harmonize all perspectives into a single implementation pathway, developers and healthcare organizations may benefit from designing context-sensitive strategies that accommodate different professional jurisdictions, workflows, and risk assumptions.

These findings have practical implications for the design and implementation of AI-enabled mental health services. First, implementation planning should begin with an analysis of professional work and care pathways, not only with an assessment of technological functionality. Second, healthcare organizations should expect the same AI application to require different governance arrangements across different settings. Third, evaluation frameworks for conversational AI should extend beyond usability and effectiveness to include organizational fit, professional accountability, and implementation sustainability. Such an approach may help move the field beyond narrow questions of acceptability toward a more realistic understanding of how AI becomes embedded in routine care.

Overall, the findings suggest that the central challenge is not whether LLM-enhanced chatbots can be introduced into mental healthcare in general, but under which professional and organizational conditions they can become legitimate, safe, and useful components of care. Future research should therefore examine how profession-specific implementation logics evolve during real-world deployment and whether similar patterns emerge across other AI-enabled clinical applications and healthcare settings.

### 4.1 Limitations

This study has several limitations. First, participants evaluated a prototype rather than a fully implemented system in routine care, and their interaction was limited to the onboarding module and the first intervention module. The findings therefore capture anticipated implementation logics rather than observed patterns of adoption, sustained use, or clinical impact in real-world settings.

Second, there was a numerical asymmetry between the two professional cohorts (*n* = 40 psychotherapists across three focus groups vs. *n* = 8 GPs in one focus group). While qualitative research prioritizes depth of insight over statistical symmetry, this imbalance means that internal variation within general practice could be explored with less granularity than within psychotherapy. Although the GP dataset demonstrated strong internal coherence and sufficient information power regarding primary care triage and signposting, the GP findings should be understood as reflecting dominant patterns within this specific primary care cohort rather than an exhaustive account of primary care perspectives.

Third, convenience sampling from a single Italian regional health authority and recruitment via a workshop explicitly addressing digital therapies and AI may have introduced selection bias. Clinicians who chose to attend may have had higher baseline curiosity or interest in digital health compared to the broader professional community. Nonetheless, participants expressed extensive critical reservations regarding clinical safety, liability, and boundary drift, indicating that the sample was far from uncritically techno-optimistic.

Fourth, detailed demographic metrics (e.g., precise years in clinical practice, personal AI usage habits) were not systematically collected due to institutional anonymity considerations, limiting our ability to assess how individual-level practitioner characteristics might intersect with professional identity.

Finally, the analysis relied on written reflections, structured group worksheets, and field notes rather than audio-recorded discussions and full verbatim transcripts. Although this design captured practice-oriented judgments produced during direct interaction with the prototype, it reduced the granularity and auditability of the analytic record and constrained the extent to which readers can independently assess the relation between interpretation and data.

Future research should examine how these profession-specific implementation logics evolve during real-world deployment, across more diverse samples, and in relation to different stages of intervention use.

## 5 Conclusion

This study suggests that the implementation of LLM-enhanced conversational agents in mental healthcare cannot be understood solely in terms of technical capability, usability, or clinical promise. Rather, implementation is shaped by how healthcare professionals interpret the role of the technology in relation to existing care pathways, professional responsibilities, and organizational conditions of practice.

Although psychotherapists and general practitioners interacted with the same technological artifact, they envisioned different pathways for its legitimate use. Psychotherapists primarily framed the chatbot as a clinician-guided adjunct to psychotherapy, emphasizing therapeutic continuity, contextual interpretation, and professional oversight. General practitioners, in contrast, positioned it as a low-threshold preventive and signposting resource within primary care, emphasizing accessibility, scope restriction, and referral pathways. These profession-specific interpretations generated different expectations regarding governance, accountability, workflow integration, and risk management.

The findings therefore indicate that the successful integration of LLM-enhanced mental health tools is unlikely to follow a single standardized implementation pathway. Instead, implementation strategies need to be tailored to the professional logics, workflow assumptions, and risk management practices of different care settings. More broadly, the study suggests that professional heterogeneity should be regarded not simply as a barrier to adoption, but as a critical source of implementation knowledge.

As conversational AI becomes increasingly embedded in healthcare, the central question is not only whether such systems can support mental health care in principle, but under which professional and organizational conditions they can become legitimate, safe, and useful components of care. Understanding these conditions will be essential for designing context-sensitive and sustainable AI-enabled mental health services.

More broadly, this study suggests that the future of AI in healthcare will depend not only on developing increasingly capable and high-performing algorithms, but also on understanding how healthcare professionals interpret these technologies and integrate them into everyday clinical practice, professional responsibilities, and decision-making processes. AI systems therefore become embedded in clinical practice when healthcare professionals assign them a socially and professionally legitimate role within existing models of care.

## Data Availability

The raw data supporting the conclusions of this article will be made available by the authors, without undue reservation.
