# Supplementary material for: How professional logics shape AI implementation in mental healthcare: a qualitative study of an LLM-enhanced chatbot
Source: Front Digit Health. 2026 Aug 31;8:1945281. doi: 10.3389/fdgth.2026.1945281 (PMC13572600; doi:10.3389/fdgth.2026.1945281)
Supplement: Supplementary file 1 [file Table1.docx]

**Supplementary Table S1.** Mapping of final themes across written reflections, group worksheets, and field notes

| **Final theme** | **Written reflections** | **Group worksheets** | **Field notes** |
| --- | --- | --- | --- |
| Shared evaluative criteria for acceptable use | Yes | Yes | Yes |
| Clinician-guided adjunct model | Yes | Yes | Yes |
| Low-threshold preventive/signposting model | Yes | Yes | Yes |
| Shared concerns about risk | Yes | Yes | Yes |
| Divergent mitigation strategies | Limited | Yes | Yes |
| Implementation requirements | Limited | Yes | Yes |
